# Workplace movement: a qualitative study on office workers’ beliefs

**DOI:** 10.1080/00049530.2024.2341695

**Published:** 2024-05-01

**Authors:** Kailas Jenkins, Jena Buchan, Ryan E. Rhodes, Kyra Hamilton

**Affiliations:** a School of Applied Psychology, Griffith University, Mt Gravatt, Australia; b Menzies Health Institute Queensland, Gold Coast, Australia; c Faculty of Health, Southern Cross University, Coolangatta, Australia; d School of Exercise Science, Physical and Health Education, University of Victoria, Victoria, Canada; e Health Sciences Research Institute, University of California - Merced, Merced, CA, USA; f Faculty of Sport and Health Sciences, University of Jyväskylä, Jyväskylä, Finland

**Keywords:** Sedentary behaviour, physical activity, qualitative, office workplace

## Abstract

**Objective:**

Physical inactivity and long hours being sedentary are well documented as behaviours that contribute to ill health. Such behaviours are prevalent among office workers, who are at risk of diseases like metabolic dysfunction, reduced bone mineral density and heart disease. Using a theory of planned behaviour belief-based approach, the current paper qualitatively explores the behavioural, normative and control beliefs held by sedentary office workers towards physical activity within the work environment.

**Method:**

Participants included 43 full-time office workers, who self-report as having a highly sedentary job and work from either a commercial office, home office, or a combination. The study used a semi-structured interview design following the theory of planned behaviour belief elicitation guidelines, and data analysed using a theoretical thematic analysis approach.

**Results:**

Participants reported advantages of stress relief, improved focus and productivity and increased positive mood with moving. Disadvantages included feelings of guilt and frustration due to work disruptions from moving more. Mixed reports for others’ approval and disapproval were identified. Office layout was a commonly identified facilitator of workplace movement, with job restrictions identified as a common barrier.

**Conclusions:**

Findings highlight the importance of workplace culture, organisational support and office layout as key considerations for future interventionalists.

## KEY POINTS

**What is already known about this topic**:

1. Physical activity and limiting sedentary behaviour are important health behaviours.
2. Office-based employees are at risk for health conditions associated with high levels of sedentary behaviour.
3. Workplace-based physical activity interventions offer a potential solution to reducing the negative impact of sedentary behaviour.

**What this topic adds**:

1. Current findings fill a knowledge gap of the attitudes and beliefs held by sedentary office workers who in contemporary society have more flexible work arrangements, thus providing greater scope of findings to inform future behaviour change programs.
2. Organisational support should be demonstrated through providing information on the benefits along with corrective feedback regarding movement within the workplace.
3. Using choice architecture to design office layouts that facilitate movement is an important consideration for both organisations and individuals.

Sedentary behaviour has been identified as one of the most consequential risks to individuals’ health and has been linked to a number of chronic conditions including metabolic dysfunction, reduction in bone mineral density, heart disease and diabetes (Hamburg et al., 2007; Narici et al., 2021; Noble et al., 2015; Park et al., 2020; World Health Organization, 2015). Of concern is the rising prevalence of sedentary behaviour, which is compounded by an increased use of technology and a decrease in physically demanding jobs (e.g., Jones et al., 2019; Kirk & Rhodes, 2011; Matthews et al., 2008; Straker & Mathiassen, 2009; Woessner et al., 2021). Previous literature investigating barriers to performing physical activity, and thus reducing sedentary behaviour, identified a lack of time as a commonly reported belief (Borowski et al., 2021; R. Rhodes et al., 2010; Salmon et al., 2003). Given a substantial proportion of time is spent at work, increasing the amount of general movement one performs in the workplace may help to increase people’s daily physical activity. Thus, countering any negative health consequences, such as posture (Dubey et al., 2019; Hemingway et al., 1997), circulation (Emanuele, 2008; Hitosugi et al., 2000) and vision issues (Loh & Redd, 2008; Mehra & Galor, 2020) from occupying sedentary jobs such as those within office’s (Higham, 2019; Mokdad et al., 2016; Noble et al., 2015; Nylander, 2016; World Health Organization, 2015). On a broader level, targeting physical movement within the workplace can also have positive effects on enhancing cognitive function leading to increased productivity (Brown et al., 2014; Kessler et al., 2003), higher engagement and job satisfaction (Arslan et al., 2019) and help reduce the burden on health care systems (Santos et al., 2023). Therefore, understanding the role that the workplace plays in influencing these health outcomes, and understanding employees’ perspectives on physical activity, is essential for mitigating these risks.

Previous literature exploring intervention strategies, such as sit-to-stand workstations, has seen positive findings in reducing sedentary behaviour (Chu et al., 2016; Hutcheson et al., 2018; Shrestha et al., 2019). Whilst these strategies provide valuable options for reducing sedentary behaviour, they do not necessarily increase light physical activity within the workplace. Previous research that has examined the effectiveness of interventions aiming to increase physical activity in the workplace has found mixed results (Dugdill et al., 2008; Malik et al., 2014). For example, in Malik et al. (2014) systematic review of workplace-based physical activity interventions, the authors found that out of the 58 studies included, less than half (*n* = 22) proved effective. The authors also discussed that there was inconclusive evidence towards which components made these interventions successful due to the heterogeneity in intervention design and delivery. High attrition rates, impractical and unrealistic activity programs and disinterest could explain the equivocal findings. Thus, establishing individuals’ beliefs towards physical movement within the workplace is important before designing and implementing programs aimed at increasing movement among sedentary office workers and may help to mitigate these issues (Malik et al., 2014).

Theories that have been applied to the understanding of physical activity behaviour include social cognitive theory (Bandura, 1989; Roberts et al., 2010), health belief model (Becker et al., 1977) and health action process approach (Schwarzer & Hamilton, 2020). Although these theories highlight the important role of attitudes (or outcome expectancies), self-efficacy and perceived risk, they lack an explicit focus on social influences towards a behaviour. Given that most workplaces are considered social environments, it is useful to understand how social influences may affect performing physical activity within the workplace environment. The theory of planned behaviour (TPB; Ajzen, 1991), in addition to attitudes and self-efficacy (akin to perceived behavioural control), offers some insight into the potential influence of social norms and important others (i.e., subjective norms) on individuals’ behaviour. However, it should be noted that broader conceptualisations of social influences, other than those prescribed by the subjective norm construct, have been advocated to understand the complexities of people’s behaviour (Hamilton & White, 2008, 2012). Broader models, such as ecological models, emphasise the impact of the social and physical environments on behaviour (Sallis et al., 2015), which might be important to take into consideration for stable environments such as workplaces. As such, previous literature has explored the environmental contexts that can instigate physical movement through the use of environmental cues (Jenkins et al., 2024). Equally important is exploring the beliefs individuals hold that drive their conscious decision-making and that may underly these behaviours. Thus, given most workplaces are social settings and the emphases on conscious processing, the TPB was adopted as the theoretical framework for the current study.

Within the TPB, intention, representing the conscious motivation to perform a behaviour, is described as the most proximal predictor of behaviour (Ajzen, 1991; Armitage & Conner, 2001; McEachan et al., 2011; R. E. Rhodes et al., 2022). Within the model, intention is determined by attitude, subjective norm and perceived behavioural control. Attitude reflects an individual’s positive and negative evaluations of the behaviour and is thought to be determined by the outcome expectancies of the behaviour. Subjective norm is described as perceptions of pressure from others to perform a behaviour, which are underlined by the expectations of people important to them either approving or disapproving of the behaviour and the individuals’ desire to comply with these expectations. Perceived behavioural control refers to the individuals perceived level of control over the behavioural production (also believed to have a direct influence on behaviour) which is thought to arise from beliefs about the extent to which factors may inhibit or facilitate behavioural performance (Ajzen, 1991). This belief basis of the TPB has been used successfully to understand physical activity behaviour across a number of population groups (Conn et al., 2003; Hall, 2022; Hamilton & White, 2007; White et al., 2007) as well as inform the design of interventions to increase physical movement (R. E. Rhodes et al., 2019).

Research aimed at understanding the determinants underlying physical activity has predominantly taken a quantitative approach (Gourlan et al., 2016; R. E. Rhodes & de Bruijn, 2013; R. E. Rhodes et al., 2009). These methods do not deliver the in-depth exploration, and thus, rich understandings, that qualitative methods can provide (Thomas et al., 2015). Studies that have used a qualitative approach to exploring influencers of physical activity have found social and physical environmental factors, such as social support, availability and accessibility of resources; and personal factors, such as physical and psychological wellbeing, as well as attitudes, to impact behaviour (Huijg et al., 2015; Kosteli et al., 2016; Ryde et al., 2020; Wendel‐Vos et al., 2007). Other studies that have explored individuals’ beliefs towards performing physical activity consistently report beliefs such as improved productivity, increased psychological health and reduced sick leave as benefits of being physically active during the workday, as well as barriers such as workload, job requirements and workplace culture (Allender et al., 2006; Cole et al., 2015; Deliens et al., 2015; Huijg et al., 2015; Kosteli et al., 2016; Ryde et al., 2020; Wendel‐Vos et al., 2007).

Niven and Hu (2018) used a qualitative approach following a TPB design to explore the attitudes and beliefs towards reducing sitting in the workplace. Their belief elicitation study was conducted on 105 commercial office workers. The authors used an online questionnaire approach to address the advantages, disadvantages, normative beliefs, feasibility and barriers towards reducing sitting in the workplace. Participants highlighted aspects such as better health as the most prominent advantage and impacts to work productivity as a disadvantage. The results also revealed a common belief that management would disapprove of a decrease in sitting time, and that a better physical work environment would be conducive to reducing sedentary behaviour (Niven & Hu, 2018). Similarly, Brierley et al. (2021) conducted semi-structured interviews using a TPB structure. The authors interviewed 10 office-based police staff in the United Kingdom on their attitudes and beliefs around reducing their sedentary behaviour within the workday. Consistent with previous literature, their findings revealed common themes around work-related tasks being seated, social norm influences of being sedentary and impact on productivity. Although these findings are helpful in understanding the motivations of office workers in reducing sedentary behaviour, they may not be generalisable given recent changes in contemporary society, with more people working from a range of environments. Research suggests there are many differences between a commercial office and home office such as air quality (Sarnosky et al., 2021), telecommunication requirements (Savić, 2020) and impacts on well-being (Xiao et al., 2021). Thus, exploring the attitudes and beliefs of office workers in a variety of workplace settings affords the identification of any differences and similarities, thereby allowing greater scope of findings which can better inform future behaviour change programs.

## Objective

To date, there has been limited qualitative research conducted on the perspectives of sedentary office workers in various work environments regarding their activity levels within the workplace using the TPB as a framework. This knowledge gap represents a missed opportunity to address critical issues that impact both employee wellbeing and organisational performance. Gaining knowledge of the subjective experience of the target population is important for understanding how to improve physical activity habits in the workday, thereby reducing sedentary behaviour often afforded by the nature of the job. Also, as employees now navigate flexible work arrangements between commercial offices, home offices, or a combination of the two, as a result of the COVID-19 pandemic (Workplace Gender Equality Agency, 2021), the dynamics of the work environment have evolved. These changes in environments highlight the importance of understanding the diverse attitudes and beliefs that individuals hold across these varied work contexts. As health habits are best formed under stable conditions, an understanding of similarities and differences across varied work environments can inform the development of future habit-based behaviour change programs that can be tailored to specific environments. To address these issues, the current study aims to qualitatively explore and identify, using a TPB belief-based approach, the behavioural, normative and control beliefs of sedentary office workers working from either a commercial office, home office, or a combination towards performing more physical movement within a workday.

## Method

### Participants

Participants were a community sample recruited via purposive sampling using social media platforms, university broadcast emails and first-year student research pool. The first-year research participant pool is a database for researchers to advertise research studies and is accessed by first-year undergraduate students. This was used as a recruitment tool as it is common for students to partake in university studies alongside full-time employment (Australian Bureau of Statistics, 2020). Study advertisements included a link to an online questionnaire where interested parties could provide their demographic data and contact details to be contacted via email to arrange the interview at the convenience of the participant. Participants were included if they were aged 18 years or older; self-described as having a highly sedentary job (sit for at least 75% of their working day (Phipps et al., 2022); and worked full time from either a commercial office, home office, or had a flexible work arrangement working from a combination of home and commercial office. Participants were excluded if they had any medical conditions that precluded them from engaging in physical activity, or if they had experienced workplace disruptions in the past two weeks due to restrictions around COVID-19 laws. Seventy-two people registered to participate by completing demographic questions and providing contact data. Of these 72, 19 did not respond to the follow-up email and 10 were excluded due to not meeting the study criteria.

The sample used for the analysis consisted of 43 adults, and participant characteristics are reported in Table 1. Participants provided informed consent upon online registration of their interest to participate, with verbal consent reobtained prior to the interviews. Participants were offered an AU$30 department store gift voucher as remuneration for their time. No prior relationships were knowingly established between participants and author KJ who conducted the interviews. Full ethical approval was granted by the Griffith University Human Ethics Committee (GU:401/2020).

**Table 1.** Participant characteristics.

		*N* = 43	Percentage of N
Gender			
	Female	28	65.10%
	Male	15	34.90%
Age			
	Range	20–64	
	Mean	38.07	
	SD	12.63	
Workplace			
	Commercial Office	16	37.21%
	Home Office	12	27.91%
	Mixed Environment	15	34.88%
Education			
	Junior School Certificate	3	6.98%
	Senior School Certificate	3	6.98%
	TAFE or Diploma Certificate	11	25.58%
	University Degree	25	58.14%
Length of Working Day (Hours)			
	Range	6–12 hours	
	Median	8 hours	
Time Spent Sedentary (Hours)			
	Range	5–10 hours	
	Mean	7.5 hours	

### Design and procedure

Interviews were conducted from March 2020 through to May 2021 in Southeast Queensland, Australia, in a residential setting with no other researchers or nonparticipants present. Participants were also asked about the stability of their workplace environment with respect to changes caused by COVID-19. The current study was guided by the COREQ (Consolidated criteria for reporting qualitative research) checklist (Tong et al., 2007). Interviews followed a semi-structured interview guide (Appendix B) consisting of open-ended questions around individuals’ behavioural, normative and control beliefs towards engaging in physical activity within the workplace environment. Physical activity was defined as any bodily movement that requires one to move from their desk as part of normal daily tasks (Armstrong et al., 1999). The interview guide was developed based on the TPB guidelines (Ajzen, 1991) as well as prior research (Hamilton & White, 2010), with feedback on question clarity received from external researchers with expertise in exercise science, psychology theory and behaviour change. The length of interviews ranged between 23 and 70 minutes, with the average taking 43 min. Forty-two of the interviews were conducted over the phone and recorded using TapeACall software and one interview was conducted using Microsoft Teams software due to a hearing impairment. Interviews were recorded and transcribed verbatim for data analysis. Participants were notified of the recording prior to the interview, and consent to participate was obtained both upon registration and verbally at the beginning of the interview. All interviews were conducted by author KJ, a female PhD candidate with honours level experience and post-graduate training in qualitative research. Participants were notified that the study was part of a larger project and would be used to form part of author KJ’s PhD thesis. KJ’s research is in behavioural science specialising in health psychology.

### Data analysis

Data analysis was conducted using theoretical thematic analysis in NVivo qualitative analysis software (Braun & Clarke, 2006, 2013; Joffe & Yardley, 2004). Data was collected, coded and analysed using an iterative process, where recruitment ceased when the data no longer added anything new to the overall analysis. Transcripts were not provided to participants for review or comment, and participants did not provide feedback on the findings. Broad categories were identified and grouped together based on the research questions. These categories were then refined into common themes within the data. Initial data coding was conducted by author KJ and to ensure coding stability, author JB reviewed the codes and deidentified interview transcripts, and author KH was consulted for any inconsistencies in coding. Themes were reviewed and refined by authors KJ, JB and KH.

## Results

Discussions about moving in the workplace were structured around behavioural beliefs, normative beliefs and control beliefs. The categories that emerged throughout the interviews appeared to be consistent between groups and thus the most salient themes are presented below (and summarised in Appendix A) around the TPB constructs with participant quotes, along with participant number, sex, age in years and workplace setting (P#, M/F, age, workplace).

### Behavioural beliefs: advantages and disadvantages

#### Advantages

When asked what participants considered as the advantages of moving during the day, across all three groups, participants responded with statements around posture like *“I get up and stretch pretty often so I don’t get stiff”* (P21, M, 25, commercial office) and *“Yes, I tend to seize up if I don’t move enough and a tend to expand or my clothes shrink very quick”* (P31, F, 57, home office). Following this, advantages identified by both the home office and combination of commercial/home office were consistent in that it helped with focus and productivity *“It helps more with the recentering and refocusing on the task”* (P36, M, 25, home office); it was energising *“Mentally, you feel, you know, a little bit more energised”* (P15, F, 57, combination of commercial/home office); that they considered it healthy *“I think just being more healthy”* (P34, F, 40, home office); and that it helped to increase mood while at work *“It helps in terms of my wellbeing. I feel a lot calmer and, you know, not anxious”* (P9, F, 29, combination of commercial/home office). For the commercial office group, responses in order of most to least common were: its energising *“You just feel, you know, more energised”* (P8, F, 50, commercial office); increases mood *“It brings your mood up”* (P1, F, 34, commercial office); helps with focus and productivity “*It increases productivity”* (P1, F, 34, commercial office); its healthy *“It increase your health”* (P39, M, 39, commercial office); good for getting sunlight *“Particularly if I go outside, get a bit of sunlight”* (P35, M, 31, commercial office); and helps for social engagement *“It’s also because on the way there you’re going with colleagues and you’re chatting and that helps pass the time as well”* (P39, M, 39, commercial office).

#### Positive experiences

Along with advantages, participants were asked to discuss any positive experiences they had experienced as a response to moving during the day. The most common responses across all three groups was that moving during the day made them feel happier and gave them a positive mood *“It makes you a lot happier and more vibrant if you move”* (P8, F, 50, commercial office), *“Keeps your mood up, and general positivity”* (P2, M, 28, home office). Following this, both the home office and combination of commercial/home office groups reported experiences of feeling relaxed or stress relief *“It’s probably relieves stress as well”* (P31, F, 57, home office); feelings of being energised *“I’m more energised to be able to continue the task”* (P15, F, 57, combination of commercial/home office); a sense of wellbeing *“Yea general sense of well-being overall”* (P31, F, 57, home office); and pain relief *“So it kind of relieves pain”* (P5, F, 41, combination of commercial/home office). Similar to these, the commercial office group reported feelings of being energised *“I feel more energized”* (P28, F, 23, commercial office) along with experiences of feeling relaxed and a relief from stress *“you feel a bit more relaxed when you get away from the computer screen and wander around for a bit”* (P30, F, 26, commercial office).

#### Disadvantages

Participants were asked if there were any disadvantages to moving during the day. Of the disadvantages identified, all three groups reported disruptions to productivity *“Your workflow being interrupted”* (P1, F, 34, commercial office), *“Breaking concentration is probably the primary one”* (P25, M, 45, home office), followed by being perceived as not working *“I think one of the disadvantages of getting up or wanting to, is that people may feel that you’re not giving your work your all, because if you’re not at your desk, with your head in those spreadsheets, they’re not getting done. I think it can potentially reflect poorly to management on performance”* (P19, F, 35, combination of commercial/home office). No disadvantage was also a common response across all three groups.

#### Negative experiences

Along with disadvantages, participants were asked if they could describe any negative experiences associated with moving during the day. The most common response across all three groups was that they did not have any negative experiences as a result from moving while at work. However, for those in the commercial office group who could identify having a negative experience, expressions of stress and frustration were commonly reported *“Probably just workload related stress”* (P32, F, 32, commercial office). For both the home office and combination of commercial/home office groups, feelings of guilt and self-loathing *“I kind of feel really guilty, like I don’t deserve to be paid to do this job”* (P6, F, 23, combination of commercial/home office); stress and frustration *“It can be frustrating, I guess sometimes”* (P17, M, 35, combination of commercial/home office); and highlights body pains *“Sometimes when I start moving my knee hurts, so it might bring out, like, different niggles in your body”* (P9, F, 29, combination of commercial/home office), where the next most common responses. One participant in the combination of commercial/home office group also reported feelings of loneliness *“If I do leave my desk at home, it’s literally just me. So the disadvantage would be like, I feel really lonely”* (P6, F, 23, combination of commercial/home office).

### Normative beliefs: approval and disapproval

#### Approval

When participants were asked who would approve of them moving more during the working day, both the home office and a combination of commercial/home office groups reported friends and family being the top supporters *“I think yes but you know friends and family would be happy about you know just get away from that computer”* (P3, F, 62, home office). This was followed by people at work which entailed colleagues at the same level as themselves *“I’m just thinking about some of my colleagues. I know that quite a few of them have also had back injuries and things like that just from sitting down. So, if I was like, oh, I’m just getting up, I’m walking around and stretching a little bit, they would approve of that”* (P14, F, 29, home office), followed by their boss or managers *“My boss, definitely”* (P13, F, 38, home office). For the combination of commercial/home office group, participants also identified their managers and colleagues. These findings were similar to the commercial office group who identified people at work: bosses and managers *“My boss has been very big on exercise and taking time to do that”* (P24, F, 26, commercial office); colleagues at the same level; and people who report to them; with one participant identifying customers *“I guess customers for what it’s worth”* (P21, M, 25, commercial office). This was then followed by friends and family. For all three groups, health professionals was reported as a group who they believed would approve of their moving during the day *“Probably my doctor”* (P8, F, 50, commercial office), and for home office and combination of commercial/home office groups, pets were also discussed *“The dog is certainly happier when I move”* (P3, F, 62, home office), *“My cat loves it when I open up my door”* (P15, F, 57, combination of commercial/home office). For the commercial office group, local businesses were also thought to be approving of moving more during the workday *“The coffee van up the road”* (P8, F, 50, commercial office).

#### Disapproval

When asked who might disapprove of moving more, the most common response across all groups was *“I can’t think of anyone”* (P2, M, 28, home office). Of the participants that did identify people they thought might disapprove, both the commercial office and home office groups reported their boss *“Oh yeah definitely the people that want me to reach my KPI’s [key performance indicators] and want me to be their little drone that does all their work for them. So upper management, 100% yes”* (P37, M, 20, commercial office), followed by colleagues *“I think the only thing would be if it impacted on the amount of work I got done. So yeah students and colleges might”* (P3, F, 62, home office). This was reversed in the combination of commercial/home office group who reported colleagues first followed by their boss. One person in the home office group also reported people who report to them *“I definitely my superior and also my subordinate”* (P40, M, 38, home office). Following this, people in the home office group identified pets as the next most common response *“Probably just my dog who gets really excited every time I go to the door and then I don’t let her out and she just sulks”* (P2, M, 28, home office), with one person in the combination of commercial/home office suggesting neighbours might disapprove if music was played too loud *“Maybe my neighbours if I have, like, a loud exercise thing”* (P9, F, 29, combination of commercial/home office).

#### Others moving more

When asked who was trying to move more during the workday, across all three groups, the most common response was people at work including colleagues at the same level, followed by bosses *“My manager that’s I know a goal that she wanted to do because feels she’s always stuck at her desk, she said that before. I think my other colleagues as well”* (P28, F, 23, commercial office). In the commercial office and home office groups, people also identified the staff who reported to them *“My boss and also my subordinates”* (P40, M, 38, home office) with one person in the home office group also identifying their clients *“I do know lots of people who would benefit from moving more who are my clients”* (P31, F, 57, home office). The next most common response across all three groups included friends and family *“My sister, who’s also working at home, she is trying to move more and my husband”* (P13, F, 38, home office), followed by the commercial office and home office groups identifying health professionals *“I followed a couple of people online who are exercise a lot and they talk about it another group could be fitness professionals or health professionals”* (P25, M, 45, home office). Across all the groups, a substantial number of participants could not identify anyone.

#### Others trying to not move more

Across all three groups most people could not identify anyone, *“I think generally people are looking for opportunities to move so they are not staying in one spot”* (P38, M, 44, commercial office). For the participants who could identify someone, friends and family were the most frequent responses. For the commercial office and home office groups, colleagues at the same level were the next most common responses *“It would be some of the other colleagues who are trying to just maybe focus on work”* (P34, F, 40, home office). One participant in the home office group also identified their clients, and one person in the combination of commercial/home office said themselves *“Well probably me because I’m just work my 7 hours and leave”* (P26, F, 52, combination of commercial/home office).

### Control beliefs: facilitating and inhibiting factors

#### Facilitating factors

When asked what factors could help people move more during their workday, across all three groups the most common response was an office set up which mainly related to distances between resources *“Having things based far apart like the car park is a walk. Um and even the bathroom is being far away”* (P9, F, 29, combination of commercial/home office). The commercial office group also reported job tasks *“I do have to walk around the whole store”* (P21, M, 25, commercial office), the home office group identified attending to pets *“Having the dog means that I need to take him out”* (P3, F, 62, home office), and the combination of commercial/home office group reported the relaxed home environment *“It’s a more casual environment being at home. So, there’s a lot more flexibility in terms of how you position yourself and posture and things like that at home course. Yeah, it’s more casual environment”* (P17, M, 35, combination of commercial/home office), as the second highest. Across all three groups, the third most common response was related to environmental factors. These related to external aspects such as being located in the city, the building having a gym, or having exercise equipment close at hand. For the home office and combination of commercial/home office groups, the next most common response related to office set up and technology *“Having my device and my phone mobile and moveable”* (P4, F, 38, combination of commercial/home office). For the commercial office group, the remainder of responses included the amount of water participants consumed resulting in bathroom breaks; getting up to speak to other staff; and planning activities into their day: *“I try and plan and activities that kind of are more active once a week”* (P28, F, 23, commercial office). For the home office group, the remainder of responses were being in a relaxed home environment; water consumption resulting in bathroom breaks; and having breaks between meetings. For the combination of commercial/home office, the remainder of responses included social acceptance: *“Just the acceptance of it and more of your colleagues doing it lends itself to you doing it without a problem”* (P26, F, 52, combination of commercial/home office); and job tasks: *“The nature of my job means that it’s not only sitting at my desk and looking at my computer, but it is going between, buildings and so forth”* (P12, M, 64, combination of commercial/home office).

#### Inhibiting factors

The most salient inhibiting factor identified by participants across all three groups was job restrictions *“The desk have to be constantly manned”* (P23, F, 33, commercial office). Lack of motivation *“Not been disciplined to do it”* (P3, F, 62, home office) and poor office set up *“The couch and coffee table is meant for leisure, not for working”* (P6, F, 41, combination of commercial/home office) were also common responses for the home office and combination of commercial/home office groups, with poor office set up relating to size being the second highest response in the commercial office group *“I wish I could measure this office for you because it’s definitely not big”* (P8, F, 50, commercial office). For the commercial office and combination of commercial/home office groups, workplace culture was the next most common response *“The kind of culture that lives within our company”* (P28, F, 23, commercial office). The remaining inhibiting factors identified by the commercial office group were restrictive work clothing, online platforms for meetings resulting in no walking to meeting rooms and feeling grounded while seated. The home office group also discussed online platforms as being an inhibiting factor to moving during the day, with one participant unable to name any inhibiting factors. The combination of commercial/home office group further identified online platforms; restricting office clothing; and not having social networks at home as inhibitors to workplace movement, with two participants unable to identify any barriers.

## Discussion

Limited research exists that qualitatively explores the beliefs of office workers across varied work environments towards moving more within the workplace. Overall, current findings showed converging evidence across participants despite workplace setting on the advantages of physical movement throughout the workday, and an acknowledgement of the health consequences of sedentary behaviour. Specifically, and consistent with previous research (Ryde et al., 2020), participants discussed that getting up and moving helped to relieve stress and tension in the body, as well as helped with focus and ability to process information. Participants also discussed that taking short breaks from sitting at the desk provided a renewal of energy and generally increased their mood. Despite the noted advantages, disadvantages and negative experiences were also discussed, the most salient across all three groups being a disruption to productivity and, as a consequence, feelings of guilt. In their paper, Cole et al. (2015) reported similar findings in that participants discussed feeling guilty for moving during paid work time when their work was desk based. This is consistent with the participants in the current study as a common disadvantage across all groups related to being perceived as not working. This belief was also addressed in previous research where participants reported feeling disruptive and awkward when they moved around within the office space (Mackenzie et al., 2019; Ojo et al., 2019).

Interestingly, participants in the current study reported mixed beliefs around the social pressures of moving during the workday (i.e., the perception that significant others would approve or disapprove of the behaviour). Managers, or bosses, were identified as being both supportive and disapproving of workplace movement. These findings are similar to those reported in previous studies (Hamilton & White, 2010). The conceptualisation of the subjective norm construct within the TPB framework could explain this mixture of findings. Previous research has shown attitudinal ambivalence to weaken the attitude-intention-behaviour relationship (Sparks et al., 2001), which might also be evident of social norm ambivalence, although further research is needed, especially for behaviours that occur within social environments. Furthermore, research exploring the social influences within this context may benefit from applying a broader framework, such as ecological models (Sallis et al., 2015), to better capture the effect of different levels of the social and environmental context on behaviour. Nevertheless, future interventions designed to use social messaging should focus on highlighting supportive messages from management and dispelling beliefs of disapproval about the utility of physical movement for cognitive function in the workplace.

Further, participants who identified their boss or manager as an “individual” were more inclined to talk about them being supportive of their movement, while those who discussed management as a group or authoritative body were more likely to perceive them as being disapproving. This finding lends to the psychology around group stereotypes, where particular characteristics are amalgamated to form an identity of out-group members as opposed to in-group members being individualised (Cooley & Payne, 2019). This perceived disapproval, along with the guilt of being perceived as not working, suggests that the role of social norms around physical activity in the workplace might be important to consider to get people to move more during the workday. Indeed, in the current study, workplace culture was discussed as a common inhibiting belief workers held towards moving more. Previous literature has found workplace culture to influence people moving more during the workday, with suggestions that organisations view the actual work being done (so not including time to move more) as billable time, have a preference for online as opposed to in-person communication, and lack behavioural modelling from senior staff (Flint et al., 2017; Morris et al., 2018; Such & Mutrie, 2017). Such workplace culture could perpetuate employee feelings of discomfort and reluctance to move more during the workday, even if it is at the determinant of workers’ health.

Job restrictions, such as the nature of the job being sedentary, high workloads and poor office set up, were other common inhibiting factors discussed. Although similar topics were discussed across all groups, participants who worked from a home office and those in the combination of commercial/home office group, discussed a relaxed home environment as a significant facilitating factor. Having a relaxed home environment meant that participants did not feel pressures to remain at their desks and had the freedom to move without causing distractions to others. Supporting this, those working from a commercial office discussed how moving during the day might cause disruptions to others. A social norms perspective, which suggests people make social comparisons and assumptions based on perceptions of what is the expected or accepted way to behave in a given setting (Dempsey et al., 2018), might explain these findings. Such perceptions may be valid or invalid, and assumptions that are derived may actually be misperceptions of the reality of the situation. In the current study, therefore, participants may be making false assumptions of their movement as being distracting to others. Such false beliefs are important for interventionalists to consider when aiming to increase physical movement within the workplace and should endeavour to diminish these misperceptions through providing corrective feedback (Dempsey et al., 2018).

Complementing the beliefs about barriers, when discussing facilitators that may help to increase movement during the workday, office layout (such as the distance between resources) was a key belief held. Participants working from a commercial office discussed how they could choose to use bathrooms further away from their desks or leave their snacks in the kitchen rather than at their desk, to increase movement. Those working from a home office, however, discussed how these strategies would only require small amounts of extra movement. Participants working from a home office or a combination of commercial/home office, often due to a response to the COVID-19 pandemic, talked about how small their working space was in comparison to working in commercial offices. Similar themes have been addressed in previous research using facilities within the environment to instigate movement (Brakenridge et al., 2018; Candido et al., 2019; Flint et al., 2017; Hadgraft et al., 2017; Hamilton et al., 2019; Jancey et al., 2016; Löffler et al., 2015). These findings highlight an opportunity for organisations to utilise choice architecture when designing the layouts of office floor plans. Designing layouts with physical movement in mind can highlight to employees the organisations' support for increasing movement during the workday (Becher & Dollard, 2016; Eisenberger et al., 1986). Although such strategies may not assist those working from an established home office, participants did discuss using the smaller home working space to incorporate small chores within their workday, such as cleaning and laundry. Practical implications from these discussions highlighted the value of using the physical environment (e.g., walking to the kitchen and bathrooms or smaller spaces), the social environment (e.g., getting up to talk to other people both personal and work-related) and taking active breaks (e.g., completing both work-related and non-work-related tasks) to instigate movement. These findings highlight the value of socio-ecological models.

Strengths of the current study include the exploration of workers’ beliefs of physical movement in the workplace using a theory-based approach, recruitment of a sample of sedentary office workers, and examination across varied workplace environments. The benefit of using a theory-based approach to a qualitative exploration is that it provides a structured approach to understanding concepts discussed, aiding in the identification of patterns and themes within the data (Hissa & Timulak, 2020). Furthermore, by using a well-established theoretical framework, researchers can draw on a dearth of knowledge to gain a deeper understanding of the relationships within the data (Hissa & Timulak, 2020). The strength of exploring different working environments is particularly important given contemporary working situations as a response to the COVID-19 global pandemic. As previous research indicates there are numerous differences between commercial and home environments (Sarnosky et al., 2021; Savić, 2020; Xiao et al., 2021), understanding the differences in attitudes and beliefs can increase the generalisability of the findings. This study, however, is not without its limitations. Interviews were conducted between March 2020 and May 2021 during the COVID-19 global pandemic, with most participants residing in Queensland, Australia. During this time, there were numerous disruptions to both the home and commercial workplace environments, such as social distancing restrictions precluding gatherings of more than 10 people. Despite attempting to account for disruptions to workplace environments through the exclusion criteria, this study did not explore lifestyle changes outside of the workplace, for example changes to family circumstances such as home schooling. Such changes may have influenced participant responses as undertaking extra responsibilities as a result of the pandemic, for example, may have induced extra stress and thus minimising the importance of physical movement (Neill et al., 2020; Phillipou et al., 2020; Ruissen et al., 2021; Shah et al., 2021). Future studies should build on these findings in times of greater workplace stability. Furthermore, the interview questions and analysis were guided by a TPB framework, and thus, findings constrained to using the model to guide the theoretical thematic analysis. Future research could extend on current findings by asking broader open-ended questions and using an inductive thematic analysis approach to gain further understandings of the influences of occupational physical activity and occupational sedentary behaviour within the workplace.

In conclusion, current findings highlight the importance of workplace social climate to people’s physical movement in the workplace. Thus, encouraging organisations to support movement from a top-down approach, and providing corrective feedback regarding peers perceptions towards moving while in the office, might prove useful to increase workplace movement. This could take the form of persuasive communications on the advantages of moving during the day and using choice architecture to design office layouts that facilitate movement.

## Credit statement

Kailas Jenkins: Conceptualization, Methodology, Investigation, Data Curation, Writing – Original Draft, Writing – Review & Editing, Project Administration.

Jena Buchan: Conceptualization, Methodology, Writing – Review & Editing, Supervision.

Ryan Rhodes: Conceptualization, Methodology, Writing – Review & Editing, Supervision.

Kyra Hamilton: Conceptualization, Methodology, Writing – Review & Editing, Supervision, Resources, Project Administration.

## Data sharing

The data that support the findings of this study are available on request from the corresponding author, KJ. The data are not publicly available due to restrictions (e.g., their containing information that could compromise the privacy of research participants).

## Appendix A Themes, Subthemes and Selected Quotes Supporting the Themes

**Table:**

Main Theme	Subtheme	Environment	Examples of Quotations From Participants
	**Behavioural Beliefs**		
	**Advantages**		
	Helps relieve body tension (Posture)	WFO	So that I don’t actually deteriorate more my body ends up being in pain (P16, F, 48)
			I get up and stretch, like, pretty often so I don’t get stiff (P21, M, 25)
			Being able to sort of get it up and stretch (P23, F, 33)
			Otherwise my back gets sore and I get like a bit wriggly (P30, F, 26)
			Get the blood flow going (P32, F, 32)
			I like to stretch my legs (P35, M, 31)
			Get up and stretch (P37, M, 20)
			My shoulders don’t feel as sore and stuff like that (P39, M, 39)
			You don’t cramp up as much (P42, M, 32)
		WFH	Yeah it’s important to get up and stretch (P3, F, 62)
			I might have preventing RSI And, um I guess you less with a bit less, um, slouching in the chair (P7, M, 36)
			It gets the oxygen to your brain and stuff to pump a bit better (P13, F, 38)
			I’m aware that there’s advantages to, um yeah, my posture And like, my core and Ah, yeah, not getting so tight if I move around more. Just like looking at a computer screen all day has been quite tiring for my eyes. Yeah. Yep. But you’re getting a break. Um, from that (P14, F, 29)
			Oh look just general health and Longevity and avoiding atrophy. And pain and misuse rather than over use. And um Yeah wastage, flexibility, blood flow, alertness (P25, M, 45)
			Yes, I tend to seize up if I don’t move enough and a tend to expand or my clothes shrink very quick (P31, F, 57)
			Getting the blood circulation that would be an advantage (P34, F, 40)
			Just to stretch good enough (P40, M, 38)
		Mixed	Gets me moving and out of my seat and actually sort of stretching (P4, F, 38)
			Physically, um, I think I need to move more because I Hopefully we’ll have less aches and pains. It’s kind of going to relieve that sort of pressure and that seizing (P11, F, 58)
			It frees, you know, frees up the blood circulation (P15, F, 57)
			Probably the main, um, reason I move will be sort of pain relief (P17, M, 35)
			Gets the blood flowing (P18, F, 53)
			Moving helps with your knee stiffness (P26, F, 52)
	Helps with focus and productivity	WFO	It increases productivity (P1, F, 34)
			I find that you work better if you move more (P24, F, 26)
			id be able to focus better (P28, F, 23)
		WFH	sometimes allows you to refocus (P7, M, 36)
			If you get up and jump on the trampoline, your brain’s kind of working a bit better (P13, F, 38)
			Oh look just general health and Longevity and avoiding atrophy. And pain and misuse rather than over use. And um Yeah wastage, flexibility, blood flow, alertness (P25, M, 45)
			It’s a good way to sort out problems. So if I’m wrestling was a problem sometimes I find the best way to I think it over is to go for a walk (P31, F, 57)
			It helps more with the recentering and refocusing on the task (P36, M, 25)
		Mixed	I’d get up and give a chance for my brain to sort of filter the information through (P4, F, 38)
			Remain more alert and, it’s good for concentration to take breaks and focus (P18, F, 53)
			It’s like moving just rejuvenates my focus (P19, F, 35)
			If I sit still, I just, I don’t think I’m as productive (P22, F, 30)
			I focus a lot when I move. So every time I feel I'm distracted, out of focus, or I'm being a bit overwhelmed, taking a walk is something that brings my focus back (P43, M, 43)
	Its energising	WFO	You just feel, you know, more energised (P8, F, 50)
			Taking a mental break (P27, F, 35)
			Better ability in having more motivation (P28, F, 23)
			I think my energy levels would Also be, would probably be improved (P28, F, 23)
			Just reinvigorate that energy (P32, F, 32)
			So it’s refreshing (P35, M, 31)
			Having those distractions breaks up the manonity (P38, M, 44)
			You don’t feel lethargic when you get up and move (P42, M, 32)
			Refresh pretty much refresh the brain. Helps with concentration (P42, M, 32)
		WFH	It’s a nice break It’s a refresher (P2, M, 28)
			Mentally, I think it can help, just, uh It’s a bit of a refresh (P7, M, 36)
			I think it will break the monotony of my day, it’s can sometimes make my mind clearer (P29, F, 29)
			Mini breaks, if you will, helps to just recharge (P40, M, 38)
		Mixed	It provides an opportunity to detach and disconnect and refresh (P9, F, 29)
			It clears my mind (P10, F, 22)
			Mentally, you feel, you know, a little bit more energised (P15, F, 57)
			Personally, I find movement energising (P19, F, 35)
			Clears my mind and take it really allows me to refresh myself and come back to the problems refreshed (P41, M, 23)
	Its healthy	WFO	The advantages would be the fact that I’m being active (P20, F, 22)
			It increase your health (P39, M, 39)
		WFH	Just general health (P25, M, 45)
			I’m sure I wouldn’t put on weight (P29, F, 59)
			Tend to expand or my clothes shrink very quick if I don’t move enough (P31, F, 57)
			I think just being more healthy (P34, F, 40)
		Mixed	Physically, I feel like I'm prioritising my health (P9, F, 29)
			It’s, like, more healthy for me (P12, M, 64)
			I feel more active (P43, M, 43)
	Increases Mood	WFO	It brings your mood up (P1, F, 34)
			I think generally for mental health (P24, F, 26)
			I probably handle situations better because I won’t have been so stuck in mobile for so long (P28, F, 23)
			Just overall mood as well, so in terms of positivity (P28, F, 23)
			I definitely feel better after I go for a walk or even just sitting outside in the sun for 15 minutes for lunch (P35, M, 31)
		WFH	I feel a lot better at the end of the day (P2, M, 28)
			Changing the mindset, so if you focusing on something, it would be a kind of a distraction or something to take your mind away from what you’re doing, and you feel actually good after that (P34, F, 40)
		Mixed	It helps in terms of my well being. I feel a lot calmer and, you know, not anxious (P9, F, 29)
	Social	WFO	Its also cause on the way there you're going with colleagues and you're chatting and that helps pass the time as well (P39, M, 39)
	Sunlight	WFO	Particularly if I go outside, get a bit of sunlight (P35, M, 31)
	**Positive Experiences**		
	Happier and positive mood	WFO	Lifting the mood (P1, F, 34)
			It makes you a lot happier and more vibrant if you move (P8, F, 50)
			It actually makes you feel a bit happy, made you feel a bit free and easy (P16, F, 48)
			More positive (P28, F, 23)
			I guess it makes me feel happy (P30, F, 26)
			It makes me feel a bit happier (P35, M, 31)
		WFH	Keeps your mood up, and general positivity (P2, M, 28)
			I usually feel really happy afterwards (P7, M, 36)
			Well, I feel a bit more happy (P13, F, 38)
			Well, so lifts my mood (P31, F, 57)
			I think exercising leads to a positive mindset (P33, F, 48)
			I think it would get me into a better mood (P34, F, 40)
		Mixed	I would probably say, uh, happiness (P6, F, 23)
			I’d say happier as well, because I don’t have the guilt of feeling like I’m not doing the right thing by myself (P9, F, 29)
			Oh, look definitely happier (P22, F, 30)
			Aside from just generally feeling positive and just calm (P41, M, 23)
			Beside the enhancing my mood. Sometimes giving me the sense that I've done some steps and exercise, which is a positive feeling (P43, M, 43)
	Stress relief/feel relaxed	WFO	Shakes off stress (P16, F, 48)
			Im probably more patient in How I approached asks (P28, F, 23)
			You feel a bit more relaxed when you get away from the computer screen and wander around for a bit (P30, F, 26)
		WFH	I guess its a release Um yeah It’s feeling calmer I guess (P3 F, 62)
			Probably less stress (P13, F, 38)
			It’s actually quite nice and quite relaxing to take a break (P14, F, 29)
			It’s probably relieves stress as well (P31, F, 57)
		Mixed	The stress is alleviated so relief I would say is a big one (P6, F, 23)
			I feel that definitely helps me feel less anxious Like it. Um, it makes me feel calm (P9, F, 29)
			The overall sense of relief (P17, M, 35)
			Aside from just generally feeling positive and just calm (P41, M, 23)
	Energising	WFO	I feel more energized (P28, F, 23)
			Feel like you’ve achieved a bit more (P32, F, 32)
			Make me feel a bit more recharged (P35, M, 31)
			Helps with being more energised (P37, M, 20)
		WFH	How shall I put it the sense of satisfaction and energising (P31, F, 57)
			It would get me even motivated to go back and work (P34, F, 40)
		Mixed	So I feel like when I move, I feel more motivated (P10, F, 22)
			I’m more energised to be able to continue the task (P15, F, 57)
			I’m more engaged with the work that I’m doing if I’ve had the opportunity to move around (P22, F, 30)
			It energises you and that you feel much better and more alert (P26, F, 52)
	Sense of well-being	WFH	It’s good for the body and the mind (P3, F, 62)
			Yea general sense of well-being overall (P31, F, 57)
		Mixed	I feel more like a human being rather than a desk monkey (P19, F, 35)
			A general feeling of well-being (P26, F, 52)
	Pain Relief	WFH	I’m a lot less stiff I noticed (P2, M, 28)
		Mixed	So it kind of relieves pain (P5, F, 41)
	**Disadvantages**		
	Disrupts productivity	WFO	Your workflow being interrupted (P1, F, 34)
			If you’re doing, like, five minutes stints I don’t think you’d get as much done (P21, M, 25)
			It kind of impacts on other things (P24, F, 26)
			Only if it was like impacting your work (P27, F, 35)
			Yes, work doesn’t get done. So by that so by getting up and moving around some days and some task. You. Just can’t get anything done because it will keep you awake for you computer (P42, M, 32)
		WFH	It’s a distraction And if you’re really involved in something it can totally derail what you’re doing (P2, M, 28)
			Any sort of interruption is really irritating (P14, F, 29)
			Breaking concentration is probably the primary one (P25, M, 45)
			Only the time consuming aspect of some of them (P29, F, 59)
			The disadvantage would be really with the workload with the deadline and also at the point like when you're focusing on something let's say I put an alarm for me at 12 to go have a snack and have a walk, let's say for an example just coming back and getting into the same concentration would be quite hard (P34, F, 40)
			Stretches every 5 minutes. That would drive me insane cause that’s going to ruin any chances of flow that I have some working on something (P36, M, 25)
			Yes it can disrupt the flow of work, the concentration (P40, M, 38)
		Mixed	If you stop with your brakes, then it’s harder sometimes for me to get back into it (P5, F, 41)
			I feel like, like sometimes I’m not getting stuff that I need to be getting done (P10, F, 22)
			Interrupt your productivity (P17, M, 35)
			Moving around could be a distraction (P26, F, 52)
			Maybe I’m like 0.1 percent less productive oh no, that’s the only disadvantage (P41, M, 23)
			Break in productivity (P43, M, 43)
	Perceived as not working	WFO	I guess there’s a fine line between moving too much and being unproductive, or perceived as unproductive (P35, M, 31)
		WFH	I feel a bit guilty. I’m not on my machine because I feel as though when I face to face on the computer that I’m kind of put it in the hours. Yeah. Now, with the teams chat and everything that cops are, I feel as though if I don’t respond straight away, people are like, Oh, where is she? She not working type of thing (P13, F, 38)
		Mixed	If it impacted my work and my career (P9, F, 29)
			I think one of the disadvantages of, you know, having to get up or wanting to is that people may feel that you’re not paying full attention giving your work your all, because if you’re not at your desk, you know, with your heading, those spreadsheets, they’re not getting done. I think it can potentially reflect poorly, um, to management, um, on performance (P19, F, 35)
	No disadvantages	WFO	I don’t really see one (P1, F, 34)
			Not at all. Hm. I don’t think there's ever a disadvantage to moving (P8, F, 50)
			Um no, no not at all (P16, F, 48)
			No (P20, F, 22)
			Mm not really (P23, F, 33)
			I don’t think there would be much disadvantage at all (P28, F, 23)
			No way (P30, F, 26)
			If I was generally, just moving for an hour every hour or something like that, just a little bursts sort of thing, I think that wouldn’t be a disadvantage (P32, F, 32)
			No (P37, M, 20)
			No I don’t. As long as its contributing to your work day (P38, M, 44)
			No, not really (P39, M, 39)
		WFH	No not really no (P3, F, 62)
			Not really (P7, M, 36)
			I wouldn’t think so (P14, F, 29)
			No, no disadvantages (P31, F, 57)
		Mixed	No I cant say that I do (P4, F, 38)
			Not particularly (P6, F, 23)
			No, not really (P10, F, 22)
			No, not at all (P11, F, 58)
			Not really, no (P12, M, 64)
			No, I don’t think that there’s any disadvantage (P15, F, 57)
			No (P18, F, 53)
			No, I don’t really have one (P22, F, 30)
	**Negative Experiences**		
	Stress and frustration	WFO	A little bit of stress (P1, F, 34)
			Probably just workload related stress (P32, F, 32)
		Mixed	It can be frustrating, I guess sometimes (P17, M, 35)
			There’s times when you just don’t have the time. Um and that can be frustrating (P22, F, 30)
	Guilt and self-loathing	WFH	I feel a bit guilty (P13, F, 38)
			If you have a deadline, I think you feel more guilty about it (P34, F, 40)
			Would then possibly be leading to further distraction and then that can perpetuate that you know self-loathing issue of I'm not getting anything done (P36, M, 25)
			With the breaking concentration frustration sometimes (P2, M, 28)
		Mixed	You’re supposed to be working, so there’s a little bit of that guilt (P5, F, 41)
			I kind of feel really guilty, like I don’t deserve to be paid to do this job (P6, F, 23)
			Guilt, getting up and walking around the office you get a massive sense of guilt (P41, M, 23)
	Highlights body pains	WFH	There’s nothing negative about moving, but apart from the injuries but they weren't so debilitating that then I was like sitting in pain (P29, F, 59)
		Mixed	Sometimes when I start moving my knee hurts, so it might bring out, like, different niggles in your body (P9, F, 29)
			The only negative is that I move and then I’m aware of, you know, the fact that I’m not 20 years old anymore (P15, F, 57)
	Lonley	Mixed	If I do leave my desk at home, it’s literally just me. So the disadvantage would be like, I feel really lonely (P6, F, 23)
	No negative experiences	WFO	None really (P21, M, 25)
			No (P8, F, 50)
			No (P20, F, 22)
			No (P23, F, 33)
			No (P24, F, 26)
			No (P28, F, 23)
			No (P30, F, 26)
			No not really (P39, M, 39)
			Nope (P42, M, 32)
		WFH	I don't see there's a negative (P3, F, 62)
			Not that I can recall (P7, M, 36)
			Nope, It’s all good (P31, F, 57)
			No (P40, M, 38)
		Mixed	No (P4, F, 38)
			Um, not necessarily (P10, F, 22)
			No (P11, F, 58)
			No (P12, M, 64)
			No (P18, F, 53)
			I don’t think so (P26, F, 52)
**Normative Beliefs**			
	**Approval**		
	Family and Friends	WFO	Um, probably my direct family (P8, F, 50)
			My partner would probably support me in moving more (P16, F, 48)
			I’m sure my friends and family would be happy with me moving on my workday (P20, F, 22)
			So just like friends, like our family (P21, M, 25)
			My mum, she's always been health conscious (P24, F, 26)
			I guess like my family (P27, F, 35)
			I think probably friends and roommates (P28, F, 23)
			Family members (P38, M, 44)
			My wife is always on my back about eating right and exercising and getting healthy, so I'm sure she would be very supportive of that (P39, M, 39)
		WFH	I think Yes but you know friends and family would be happy about you know just get away from that computer (P3, F, 62)
			I guess friends and family, um, because they probably care about the health aspects of it (P7, M, 36)
			My family, yes, my husband and mother (P13, F, 38)
			My partner would approve (P14, F, 29)
			Yea well I think like my immediate family would like to spend more time with me (P25, M, 45)
			My kids are happy about my progress and so are the swimming teachers I'm friends with (P29, F, 59)
			My husband, hes probably the most one that encourages that (P33, F, 48)
			I think a first of all it would be my partner, then my family in general as they expect me indirectly just to be healthy (P34, F, 40)
			My spouse (P40, M, 38)
		Mixed	Ah, especially my partner (P5, F, 41)
			So there’s sort of like a managers and supervisors and then family and friends (P6, F, 23)
			Ah, my family, my partner, my friends (P9, F, 29)
			My partner would be one, so I'm not so drained and grumpy (P10, F, 22)
			Ah, my partner and my family (P11, F, 58)
			Ah, my husband (P15, F, 57)
			Ah, well my kids and my wife primarily (P17, M, 35)
			My partner, they notice a difference in my attitude when I move. I think my friends and family would feel the same as well (P22, F, 30)
			In terms of who would feel positive, my GP, my family, just getting up off my ass from sitting on the computer all day (P41, M, 23)
			My wife, I would say my parents and, my wife. Those are three people who are extremely happy (P43, M, 43)
	People at work	WFO	The new employees (P1, F, 34)
			The staff members I would be in charge of (P21, M, 25)
			I guess customers from what it’s worth (P21, M, 25)
			My boss, they would probably be like, oh its good that you get up and move (P21, M, 25)
			Um, supervisors and the manages (P23, F, 33)
			My boss has been very big on exercise and taking time to do that (P24, F, 26)
			Probably in my direct manager and sorry my colleagues would approve as well (P28, F, 23)
			I think sort of my team in general like we all really benefit from when we do sort of run into each other briefly around the floor like because we can because we are in our own spaces (P32, F, 32)
			I like to think the leadership team would support the fact that we get up and walk more, within reason (P35, M, 31)
			id say the main group of people that I typically work with (P37, M, 20)
			I guess generally that would be your work colleague (P38, M, 44)
			So my manager, that’s really the main person, I mean probably my colleague as well (P39, M, 39)
			My subordinates and my boss, cause if you're getting up and moving your talking to your crew, you know what's going on and that helps with the situational awareness of what's happening around you (P42, M, 32)
		WFH	Um my manager is very health conscious I think she’d be very happy about me moving more during the day (P2, M, 28)
			Probably my coworkers (P7, M, 36)
			My boss, definitely (P13, F, 38)
			I’m just thinking about some of my colleagues. I know that quite a few of them have also had, um, you know, back injuries and things like that just from being sitting down. So if I was like, Oh, I’m just getting up, I’m walking around and stretching a little bit. They would approve of that (P14, F, 29)
			Collegues, as well (P25, M, 45)
			My supervisor who keeps on moving herself, and other thing is, She keeps on telling me to take good care of myself (P34, F, 40)
			Yeah colleagues or people on the same level as me in the organisational chart (P40, M, 38)
		Mixed	Some of my director and boss (P4, F, 38)
			I would probably say my supervisor and my direct, um, line of reporting my management (P6, F, 23)
			Ah, my family, my partner, my friends, my work colleagues (P9, F, 29)
			So I'm assuming that colleagues (P11, F, 58)
			Colleagues, if I have a question, I’m much more likely to just walk across the floor and ask someone (P19, F, 35)
			I know my colleagues would definitely feel the same (P22, F, 30)
			I would say managers (P26, F, 52)
			If my boss wants me to do something he would expect me to move and do it. That’s it. Those are people who come to mind and are actually happy (P43, M, 43)
	Health professionals	WFO	Probably my doctor (P8, F, 50)
			Work Health Safety team (P35, M, 31)
		WFH	So I guess health authorities and the massuse would really ah encourage me to move more (P3, F, 62)
			My coach, my Muay Thai coach (P33, F, 48)
		Mixed	So, um, I think, uh, one person HR department is always trying to get us to get up and move more (P19, F, 35)
			I’m not sure if But yeah my doctor might approve (P26, F, 52)
			In terms of who would feel positive, my GP, my family, just getting up off my ass from sitting on the computer all day (P41, M, 23)
	Pets	WFH	The dog is certainly happier when I move (P3, F, 62)
			Other people who would love me to take more breaks is the dogs (P13, F, 38)
			So I also have two dog. Ah. If I get up and walk around like they’re probably really happy because they’ll be running around the yard or for the other one a couple of times (P14, F, 29)
			My dog he probably like that more (P36, M, 25)
		Mixed	My cat loves it when I open up my door (P15, F, 57)
	Other	WFO	The coffee van up the road (P8, F, 50)
		WFH	Anyone who has any sort of chronic pain issue (P2, M, 28)
	**Disaproval**		
	People at work	WFO	My boss would disapprove of me moving more and probably all the executives if they were trying to get a hold of him and couldn’t get a hold of me (P16, F, 48)
			Definitely my supervisors (P27, F, 35)
			Maybe my coworker? Cos if I leave the room then something happens, she’s alone or she can’t leave the room (P30, F, 26)
			I do think that like if I'm up having a chat, and its my thoughts, but you know is my boss thinking. You know. They’re having a chat and I should be doing work or do you know if my boss is in ear shot I don’t want him to think that I'm being slack or I'm not doing my job (P32, F, 32)
			I think definitely like so because were all involved in project delivery project. The sort, your workload is never the same like the project to go through peaks and troughs. So definitely think that people are probably going for a peek are more disapproving of it (P35, M, 31)
			Oh yea definitely the people that want me to reach my KPI’s and want me to be their little drone that does all their work for them. So upper management, 100% yes (P37, M, 20)
			Maybe my manager again. Just depending on how disruptive it is for the work (P39, M, 39)
		WFH	I think the only thing would be if it impacted on the amount of work I got done So yeah students and colleagues might (P3, F, 62)
			My boss would like me to be chained to my desk 24/7 (P25, M, 45)
			I definitely my superior and also my subordinate. Superior thinks that I'm slacking off non productive activity. Subordinate would think that I am trying to walk around their desk to see, to check on them. Even though really I couldn’t care less (P40, M, 38)
		Mixed	I think the work colleagues, only if the perception was that I’m slacking off (P9, F, 29)
			Yes. Management (P19, F, 35)
			I think there’s always the fear of, like, especially people in leadership positions in the workplace, not appreciating the value of giving people the opportunity to move around (P22, F, 30)
			If co-workers were possibly distracted or disturbed then maybe but yea (P26, F, 52)
			My manager and other colleagues (P41, M, 23)
			Maybe maybe my neighbours if I have, like, a loud exercise thing (P9, F, 29)
	Pets	WFH	Probably just my dog who gets really excited every time I go to the door and then I don’t let her out and she just sulks (P2, M, 28)
	No one	WFO	No (P1, F, 34)
			No (P8, F, 50)
			No (P20, F, 22)
			No (P21, M, 25)
			No (P23, F, 33)
			No (P24, F, 26)
			I don’t think so (P28, F, 23)
			No (P38, M, 44)
			No (P42, M, 32)
		WFH	I can’t think of anyone (P2, M, 28)
			Probably not (P7, M, 36)
			No (P13, F, 38)
			No (P14, F, 29)
			No, no body (P29, F, 59)
			No (P31, F, 57)
			No (P33, F, 48)
			I don’t think so (P34, F, 40)
			I would highly doubt that (P36, M, 25)
		Mixed	No (P4, F, 38)
			No (P5, F, 41)
			No (P6, F, 23)
			No (F10, F, 22)
			No (P11, F, 58)
			No (P12, M, 64)
			No (P15, F, 57)
			No (P17, M, 35)
			No (P18, F, 53)
			No (P43, M, 43)
	**Others Moning More**		
	People at work	WFO	I think the staff members (P21, M, 25)
			Doing the sedentary, um, life style of sitting down for 8 hours I would say Yeah coworkers (P23, F, 33)
			My manager that’s I know a goal that she wanted to do because feels she's always stuck at her desk, she said that before. I think my other colleagues as well (P28, F, 23)
			I know one of my colleagues. Just makes a habit of it, but I think he’s always done that. You know for a long time like you make an effort to do it, and I notice him up and about, Regularly and not necessarily talking to anyone but just up and moving (P32, F, 32)
			All the coworkers who have signed up to the Kokoda (P35, M, 31)
			There's this one girl that I talk to a lot in another department. She’s like all about, she's super healthy super fit, she's always wanting to move around cos she works down by the pool (P37, M, 20)
			We started that walking at lunch time because there were a couple of us that were you know bit to wanting to do a bit more during The day (P39, M, 39)
			Yea my subordinates. Pretty much all pretty much all of us in the workplace try to move more or try to move as much as we can (P42, M, 32)
		WFH	Some of my colleagues I don’t really know Um the some have made noises about you know being stuck at home and really have to think about getting up and moving because there’s no incidental movements like you don’t don’t be like popping over to someone’s office or going to a coffee meeting or something like you normally would. So it’s been a bit of a focus on that (P2, M, 28)
			Um yeah colleagues Talk about it So yeah I think it’s something we’re aware of in terms of you know health in the workplace (P3, F, 62)
			Say just in general people around the office are more cognitive of the need to stand up during the day and say just colleagues and people in general that have sedentary jobs (P25, M, 45)
			My business partner is the only and she’s in Hong Kong and she will go for a walk or a big walks like I guess we call them hikes (P29, F, 59)
			I do know lots of people who would benefit from moving more who are my clients (P31, F, 57)
			Yes, I say my colleagues and some of my friends (P34, F, 40)
			My boss and also my subordinates. Yea the reason for that the idea that they present therefore that they remain relevant (P40, M, 38)
		Mixed	I would say my team (P6, F, 23)
			Like my big boss in Brisbane. They’re trying to implement, um, like, stand up desks (P10, F, 22)
			Some people here at work (P12, M, 64)
			Yeah my team there (P15, F, 57)
			That would be my boss (P18, F, 53)
			Yeah, definitely the agents on the phone (P19, F, 35)
			Uh, all of my colleagues are definitely trying to move more (P22, F, 30)
			A couple of very few co-workers (P41, M, 23)
	Friends and Family	WFO	Yeah, my mother’s trying to move more (P8, F, 50)
			My partner definitely (P16, F, 48)
			Yeah. My sister works in the office, and I don’t think she gets much time to get up and move (P21, M, 25)
			My partner thinks the same thing and definitely my family members. So direct family mum dad brother and sister they all think its awesome (P35, M, 31)
		WFH	My sister, who’s also working at home, she is trying to move more and my husband (P13, F, 38)
			So I know, um, my partner, instead of driving or getting the bus and train to work, he’s been cycling to work (P14, F, 29)
			Yes, I say my colleagues and some of my friends (P34, F, 40)
		Mixed	My mom but she works in a call centre (P4, F, 38)
			My friend is actually an exercise physiologist (P5, F, 41)
			My daughter, who was working at home for a time, she’s also had aches and pains. And then my partner (P11, F, 58)
			My wife. Yeah. She’s Yeah, she’s good with it (P17, M, 35)
			My partners the same. Um, he makes a considered effort to get up and move around during the day (P22, F, 30)
			Yeah my mother. She tries her best to increase her movement (P43, M, 43)
	Health professionals	WFO	There’s always the Instagram culture, but the Instagram influences. Yep (P24, F, 26)
		WFH	I followed a couple of people online who are exercise a lot and they talk about it another group could be fitness professionals or health professionals (P25, M, 45)
			My Muay Thai coach (P33, F, 48)
	No one	WFO	I don’t think so no (P1, F, 34)
			Not to my knowledge (P20, F, 22)
			No (P27, F, 35)
			I really don’t think so (P30, F, 26)
			No (P32, F, 32)
		WFH	Not really anyone that I can think of (P7, M, 36)
			I don’t know it’s not really a topic of discussion (P36, M, 25)
		Mixed	No (P9, F, 29)
	**Others Trying Not To Move More**		
	Family and Friends	WFO	Umm probably my whole family, yea they don’t, like I actually had to talk to my mum about she had been, like during covid, she had been at my sisters for like 16 weeks or how ever long it had gone and she was literally in the house for all of that time (P16, F, 48)
			My brother’s a landscaper. I bet he’d love to sit down more (P21, M, 25)
			My friends who work in hospitality (P28, F, 23)
		WFH	My parents, my children (P25, M, 45)
			Oh my son. Oh that’s not fair he does do his work out. But then he sits at his computer even longer than I do. And my parents (P29, F, 59)
		Mixed	Yes, my son (P11, F, 58)
	People at work	WFO	Probably my colleague in the room that I'm in. A lot of People actually they eat lunch at their desk, they don’t like to leave for anything (P30, F, 26)
		WFH	Also my clients yeah, so I think most people would rather wish they didn’t have to move as much as they do (P31, F, 57)
			It would be some of the other colleagues who are trying to just maybe focus on work (P34, F, 40)
	Other	Mixed	Well probably me because I’m just work my 7 hours and leave (P26, F, 52)
			I think there’s always people who are going to be happy just to sit at their desk all day (P19, F, 35)
	No one	WFO	No (P8, F, 50)
			No (P20, F, 22)
			No (P23, F, 33)
			No (P24, F, 26)
			No (P27, F, 35)
			No (P32, F, 32)
			Its not really a conversation I've had with any people. I don’t know of anyone who would say id rather sit down. Unless is that it was hot day outside and I said I was going to walk around the block in which case I would expect that a couple of people, so I’m not. (P35, M, 31)
			No (P37, M, 20)
			I think generally people are looking for opportunities to move so they are not staying in one spot (P38, M, 44)
			No (P39, M, 39)
			No (P42, M, 32)
		WFH	No (P3, F, 62)
			No (P7, M, 36)
			No (P13, F, 38)
			No (P14, F, 29)
			No (P33, F, 48)
			No (P36, M, 25)
			No (P40, M, 38)
		Mixed	No (P4, F, 38)
			No (P6, F, 23)
			No (P9, F, 29)
			No (P10, F, 22)
			I wouldn’t think deliberately (P15, F, 57)
			No (P17, M, 35)
			No (P18, F, 53)
			No (P22, F, 30)
			No (P41, M, 23)
			No (P43, M, 43)
**Control Beliefs**			
	**Enabling Factors**		
	Office set up	WFO	Walking up one flight of stairs from the car park to the lift (P1, F, 34)
			Pick something up off the printer (P8, F, 50)
			The bathroom is kind of like a distance away (P23, F, 33)
			The printer is a little bit of a distance, probably about 10, 15 steps. Oh the kitchen is further than that, and then the bathroom further than that (P24, F, 26)
			The bathroom is up a flight of stairs (P27, F, 35)
			The bathroom and the kitchen are on the other side (P30, F, 26)
			Definitely having the kitchen at on the other side of the building and I purposefully leave my lunch in the kitchenette which means I have to walk a little bit further. When I have team meetings I do try and walk over to the shared office space. Also quite often I will go to the bathroom that are furthest away so I can get a bit more of a walk (P35, M, 31)
		WFH	I have to go up and down stairs to get to food and drink (P13, F, 38)
			Not having a lot of clutter in the room with me so I can move around (P25, M, 45)
			I live alone if I want to get up and pace the floor for some reason or just get up and move or do something silly like dance. If I’m celebrating something, the kind of the kind of movements that would cause people in the workplace to look at me sideways I can do at home without an ounce of embarrassment (P31, F, 57)
			I have my stairs that I run up and down them (P33, F, 48)
			The kitchen and toilet are maybe 20 steps away (P34, F, 40)
			Having the kitchen downstairs (P36, M, 25)
			The environment itself, just not being boxed in, having an easy access to outside if I need to walk out I don’t have to go between cubicles and filing cabinets. A straight in straight back kinda rout. Open access. I suppose having like kitchen and stuff downstairs also, You get that bit of movement walking up and down the stairs as well (P40, M, 38)
		Mixed	We have to walk downstairs for lunch and there’s a the lunch spot is quite a walk from where I’m based (P5, F, 41)
			My lounge is quite spacious. So like I said, if I do decide to get up and move my laptop to the kitchen counter, I can (P6, F, 23)
			Having things based far apart like the car park is a walk. Um and even the bathroom is being far away (P9, F, 29)
			Well, the fact that I’m in the room by myself, um, makes it really easy for me to get up and do things without being self-conscious. being at home means that Ah, just ah. Being able to open the door and just walk outside and check the mail (P15, F, 57)
			I’m lying on the floor because I’ve got space (P17, M, 35)
			It’s a bit of a walk from the kitchen to the office (P18, F, 53)
			The fact that our office opens out straight onto the main road, and you’re able to access the outside. Open plan layout, And we also have numerous exit points outside the office that helps as well (P26, F, 52)
			I have a lot of space at home to move around (P41, M, 23)
			The stairs would also help as well (P43, M, 43)
	Job tasks	WFO	I do have to walk around the whole store (P21, M, 25)
			We have all our gymnastics equipment that lives on site so, frequently if we have to go do a check on that (P28, F, 23)
			Someone’s needing a certain something from my area than I'm needed to run that thing to them (P30, F, 26)
			Having subordinates that I got to go out to check (P42, M, 32)
		Mixed	The nature of my job means that it’s not only sitting at my desk and looking at my computer, but it is going between, buildings and so forth (P12, M, 64)
	Pets	WFH	My partners dog is playful sometimes (P2, M, 28)
			Having the dog means that I need to take him out (P3, F, 62)
			I’ll take the dog out do something and then come back and try and focus (P36, M, 25)
	Relaxed home environment	WFH	If I am really procrastinating on something, I might stand up and decide that I’m going to unpack this box of books or I’m going to fix this bookshelf (P36, M, 25)
			My boss Can’t see me (P40, M, 38)
		Mixed	Keeping up to date with the washing is the best (P11, F, 58)
			There is a little bit more freedom at home (P12, M, 64)
			It’s a more casual environment being at home. So there’s a lot more flexibility in terms of how you position yourself and posture and things like that at home course. Yeah, it’s more casual environment (P17, M, 35)
			Doing like things like the house work (P19, F, 35)
			The mindset I think as well you’re a lot more comfortable in the own house. You’re also not in office ware (P26, F, 52)
	Broader Environment	WFO	We do have a lake like a little lake just outside of work (P1, F, 34)
			I suppose being in the city because you can go do things during the day (P24, F, 26)
			I try to do some personal stuff in Brisbane (P38, M, 44)
		WFH	I mean we’ve got all the gym equipment (P29, F, 59)
			In our building we have a gym which is fully equipt so that encourages me to exercise and downstairs we have a really large pool. I have my yoga mat, I have weights, I have my skipping rope I have a hula hoop, a weighted hula hoop (P33, F, 48)
		Mixed	I have one of those exercise balls instead of chair. So during lunch time. So we, um my other colleagues go to the gym when I just go to the pool (P5, F, 41)
			Best things about working at the Gold Coast campus was doing the occasional lunch time yoga class. Ah, or the occasional, um, because my building was across the road from the swimming pool (P11, F, 58)
			My yoga mat is here, and exercise ball that work won’t let me have is here. I’ll jump on the exercise bike at lunch time when I’m working from home (P19, F, 35)
			We have a beautiful office with a lot of windows. Um, so you can sort of see right down to the beach. So, yeah, it’s really, really nice. Um, and sometimes that’s enough to kind of get you moving (P22, F, 30)
			We have a small basketball court outside so I can just do some shooting (P43, M, 43)
	Water consumption	WFO	I make sure that I drink a lot of water, two reasons, it means I go to the kitchen a lot and I go to the toilet a lot (P16, F, 48)
			I drink so much water, meaning I pee a lot (P32, F, 32)
		WFH	I use a cup for my water. So I have to get up and refill it all the time (P7, M, 36)
	Technology	WFH	Having a headset (P13, F, 38)
			Having a standup desk (P25, M, 45)
		Mixed	Having my device and my phone mobil and moveable (P4, F, 38)
			Having headphones connected to your phone or even when there's a collaborate session with students. You can turn your camera off and just walk around (P5, F, 41)
			My set up is not very economic. So it makes it easy for me to walk away because I sometimes just like, physically need to stand up because my chair is shit (P22, F, 30)
			I use a lot of the whiteboard, so I get up to do that (P43, M, 43)
	Social acceptance	Mixed	The main policy that’s being driven at work by HR is stretching (P19, F, 35)
			There’s a lot of, like, well-being kind of paraphernalia on the walls and stuff. So there’s encouragement to go and do walking meetings (P22, F, 30)
			Just the acceptance of it and more of your colleagues doing it lends itself to you doing it without a problem (P26, F, 52)
	Other	WFO	I try and plan and activities that kind of are more active once a week (P28, F, 23)
			Ill walk over to them if I need to speak to another staff member (P28, F, 23)
		WFH	Just having breaks between meetings. Yeah, if I’ve got a lot of meetings, ill schedule time away from my desk (P13, F, 38)
	**Inhibiting Factors**		
	Job restrictions	WFO	We’re actually restricted now because you can’t actually go anywhere because it is just me. Also the role itself (P8, F, 50)
			The amount of work or the type of work that I have to do in the space that I have to do it (P16, F, 48)
			Having to be sitting down for the job (P21, M, 25)
			The desk have to be constantly manned (P23, F, 33)
			I still need adhere to the same rules and be on the same cell phone line (P27, F, 35)
			If I’m alone I can’t leave (P30, F, 26)
			Its just me in the office now so I sort of need to be here during opening hours and the nature of my work is just mostly on the computer (P32, F, 32)
			We technically can only get up and move for our break which is every 6 hours (P37, M, 20)
			The nature of the work is seated (P38, M, 44)
			The work is, just revolves around the computer (P39, M, 39)
			Constant paperwork and constant administration, phone calls on the phone, people wanting to have private conversations, so you obviously got to sit down and listen to that (P42, M, 32)
		WFH	You do have to be glued to your desk because, you know, a phone call could come through at any moment (P7, M, 36)
			Ah, probably deadlines and trying to get through a lot of work, makes it difficult (P13, F, 38)
			Ahh having deadlines (P25, M, 45)
			I don’t have time to when I’m working fully working (P29, F, 59)
			I guess if I was a bit more, like really under pressure with deadlines or something, I'm not going to be as inclined to be taking break (P36, M, 25)
		Mixed	Time and very task orientated work (P10, F, 22)
			I think one of the things is workload (P11, F, 58)
			The main thing is, is the nature of the work (P12, M, 64)
			I guess the thing that prevents me is just the work (P15, F, 57)
			Look, I just think being tied to the phone really matters quite a lot (P19, F, 35)
	Lack of motivation	WFH	Not been disciplined to do it (P3, F, 62)
			I would probably say the biggest inhibitor to me getting up and moving is just myself and my own motivation. Or lack there of (P14, F, 29)
			My mind is the only thing (P33, F, 48)
			Being too immersed in doing the work (P34, F, 40)
		Mixed	It’s easier to be to be lazy at home (P9, F, 29)
			I’m pretty lazy when it comes to exercising. So psychologically not preparing myself to be able to have those breaks. It’s hard to leave the work behind (P15, F, 57)
			The boundaries aren’t as clear, so I can find myself working for far longer (P22, F, 30)
			If hes home like he is now for instance, definitely I would stay up more just because it would be less distracting for me (P26, F, 52)
	Poor office set up	WFO	I wish I could measure this office for you because it’s definitely not big (P8, F, 50)
			Having a small office space (P24, F, 26)
		WFH	Going to the kitchen or to my bathroom. The distances are so much shorter (P14, F, 29)
			Everything is very compact, everything is very close (P29, F, 59)
			Everything is so close so it’s a shoe box of a flat (P31, F, 57)
		Mixed	The couch and coffee table is meant for leisure, not for working (P6, F, 41)
			At home everything is so close together, it’s a very small apartment (P9, F, 29)
			The bathroom is quite close (P41, M, 23)
			Probably that awkward way the building is set up like there’s no open space in front of you. Have to either go down the stairs or you have to cross to the second building so you can then find an open space to walk (P43, M, 43)
	Workplace culture	WFO	Yep, it all stems from like workplace culture. its just how things have been for years, and they continue to do it, but they don’t think about the side effects (P27, F, 35)
			The kind of culture that lives within our company (P28, F, 23)
			No real motivation by higher up (P37, M, 20)
		Mixed	My boss is in the office and she’s next door. So she kind of expects me to be there when she there (P9, F, 29)
			People sort of expect you to be framed during meetings (P17, M, 35)
			I asked if I could use a fitness ball to sit on, um, which was recommended by physio. However, they said no, Um, because it’s not recommended from a HR from a workplace health and safety record (P19, F, 35)
			I’m in a team full of clock watches and they notice when you get up and go for a walk (P41, M, 23)
	Restricting clothing	WFO	I wear heals (P20, F, 22)
			I also dress corporate to work so my attire plays a big role (P28, F, 23)
		Mixed	You’re not really dressed to move around a lot. So heels and office clothes (P26, F, 52)
	Online platforms	WFO	Meetings that we used to have face-to-face or all done via teams (P39, M, 39)
		WFH	You just, like, log off one meeting and then go onto the next one. So you’re not getting up (P14, F, 29)
		Mixed	There’s no longer the meeting rooms to physically go to (P11, F, 58)
			The interaction with people is less organic at home and actually does require you to be sitting at a desk to interact with them rather than moving (P19, F, 35)
	Other	WFO	I probably feel grounded when I'm seated (P16, F, 48)
		Mixed	I don’t have social networks here at home (P5, F, 41)
	None	WFH	Actually no, not really (P40, M, 38)
		Mixed	I can’t really think of any (P18, F, 53)

## Appendix B Interview Guide

**Interview Protocol/Questionnaire**

**Table:**

**Interview# ______________** **Time** ______________ **Date/Day ______________** **Location ______________**

**Pre-Interview Instructions**

- Email the relevant information sheet
- Confirm participant is happy to participate and commence the interview
- Read the demographic survey to the participant and fill out the appropriate responses

**Procedure**

***Introduction and Purpose of the Interview***

- Introduce investigator and affiliation.
- Thank participant for agreeing to take part in the interview.
- Inform participant that the interview will be based around a series of open-ended questions to understand more about their beliefs, attitudes, barriers and potential opportunities associated with increasing the amount of general physical activity within their working day.
- Inform the participant that the information they provide may be used to inform the development of material that will be used for an intervention aimed to increase physical activity in sedentary office workers.
- Let participant know you are grateful for their time and encourage them to participate in the interview as much as possible and to answer questions as honestly as they can.
- Inform participant that the interview will last for approximately 30 minutes.

***Interview Rules – Inform participate of the following …***

- Role of the Interviewer
  - My role is to facilitate the interview by asking questions and, if needed, asking for clarification or further information. My role is to also to guide the interview so we do not deviate from the topic of discussion.
- Permission to Record Interview
  - As per your information sheet, the interview session is being audio-recorded. This will allow me to focus on what you are saying and not rely on my memory.

- Rights of Participants Regarding Participation
  - The responses you provide are confidential and anonymous.
  - Your participation is voluntary and you are free to withdraw at any time without penalty.
  - Any information obtained from this study will be deidentified so that no individual participant can be identified.
  - All information including audio tapes and transcribed data will be securely stored and accessible only to the researchers.
  - Upon completion of the study, all audio recordings will be destroyed.
- Rights of Participants Regarding Interview Discussion
  - There is NO right or wrong answers so please respond as honestly as you can.
  - I really value your opinion on this topic so please feel free to share your experiences as openly as possible.
  - Please try to speak as clearly as you can to help with the audio recording.
- Are there any questions?
- Do I still have your permission to tape record this interview?
- Do you have any concerns or would like to change your mind about participating?

**Interviewer Script**

Hi [*name*] its Kailas from Griffith University, how are you this morning/afternoon?

I'm calling in regards to the interview for the “increasing your movement in the workplace” study, is this still a good time for us to talk?

Ok so before we begin ill run you through the process and give you a chance to ask any questions that you might have.

So firstly we will go over a few consent points before jumping into the actual questions. You might find that some of these questions are quite vague so there’s no right or wrong answers, the point is for me to get an understanding of what your day is like. And just to let you know I’ll be jotting down some notes as we go.

How does that sound? Great, do you have any questions at this point?

Ok awesome, so before we get into it, you should have received an email with a copy of the study information attached.

This is the same information that you would have read when you registered for the study.

No? – Ah not a problem I will email that through to you now so you have a copy. Can I just confirm your email address is …

Yes? – Ok great.

So I’ll read you the verbal consent points now and at the end of them I’ll ask you if you agree to participate, if at any point you have any questions just let me know.

1. I had the participant information sheet provided to me and I understand the information. The research project has been fully explained to me and I have had all my questions answered. I know that I may ask for more information about the project as it goes on.
2. I understand that taking part in this project is voluntary, and that I may stop taking part in the project at any time.
3. I understand that the interview will be audio-recorded and audio-tapes will be transcribed and analysed. All audio-recordings will be erased after the project concludes.
4. I understand that all information will be treated in the strictest confidence and used for research purposes only. I understand that I will not be personally identified in any reports from this project.
5. I assign and waive all claims to patents, commercial exploitation, property or any material or products which may form part of or arise from this study.
6. I understand that this research will comply with the National Health and Medical Research Council’s National Statement on Ethical Conduct in Research Involving Humans and with the privacy policies of Griffith University.
7. I understand that this study has been approved by the Griffith University Human Research Ethics Committee. I understand that if I have any questions about the ethical conduct of this study, I can contact the Griffith University Research Ethics Officer on (07) 3735 4375.

**Do you give your verbal agreement to participate in this research study?   Yes   No**

Name of Participant:———————————————————————   Date:——————

Name of Interviewer:——————————————————————   Date:——————

Signature of Interviewer:————————————————————    Date:——————

Great, so now that’s out of the way we can get into the actual discussion. So I have here that you currently:

- work-from-office
- work-from-home
- mixture of work-from-office and work-from-home

Was your this affected by COVID at all?

Can you tell me a little bit about the details of your workplace?

– How you get to work?
– Describe your workspace to me?
– What is the social environment like in your workplace?

So with regards to moving more during your day: What do you see as the **advantages**?

– What **positive** experiences or feelings do you think would result from you moving more during your workday? (e.g. excited, happy, glad, satisfied, proud)
– What do you see as the **disadvantages** of you moving more during your workday?
– What **negative** experiences or feelings do you think would result from you moving more during your workday? (e.g. angry, guilty, ashamed, sad, disappointed, worried)

With regards to the social aspects of moving more during your day:

– Who are the individuals or groups, that are important to you, who you feel would **approve** of you moving more during your workday?
– Who are the individuals or groups, that are important to you, who you feel would **disapprove** of you moving more during your workday?
– Are there any individuals or groups important to you who themselves are trying to move more during their workday?
– Are there any individuals or groups important to you who are not trying to move more during their workday?

In regard to your physical work environment:

– What are the factors and circumstances that would make it **easier** for you, or would **enable** you, to move more during their workday?
– What are the factors and circumstances that would make it **difficult** for you, or would **prevent** you from moving more during your workday?

Thank you for all we have discussed so far. Now I would like to talk about the routines you have when you’re at work.

– Describe to me what a typical work day would be like for you?
– Would you consider some of these things that you do to be more regular and routine than others?
– When thinking about your workday, what things could you use as reminders to move more?
– So during your day, what are the types of things that you get up from your desk to do?

If you had to pick 3 different actions that you perform regularly at the office, what would they be?

(1) Receiving phone calls
(2) Printing
(3) Disposing of rubbish in bin

If you had to pick 3 different actions that you perform regularly at your home office, what would they be?

(1) Receiving phone calls
(2) Teleconference meetings
(3) Making tea, coffee or water

*For mixed home/office workers*

– Think about both your home office and work office, what are some of the similarities between the environments?
– Do you do any chores or outdoor activities during your day? → Can you expand on this for me

**The purpose of our discussion today was to gain knowledge from office workers about their views on moving more in the workplace, such as benefits and barriers. Do you think that we have missed anything important in our conversation?**

Before I conclude, I would like to summarise what has been discussed here today and check that you agree with what I have understood from our discussions. The main things I have gathered from the interview today are …

Do you agree with what I have said? Do you have any questions or comments?

Thank you SO MUCH for taking the time to participate in this interview. I really appreciate your participation and effort. If you have any questions, please feel free to contact a member of the research team – the researchers email address and phone number are on your information sheet.
